# Self-reported data: a major tool to assess compliance with anti-malarial combination therapy among children in Senegal

**DOI:** 10.1186/1475-2875-8-257

**Published:** 2009-11-17

**Authors:** Aurélia Souares, Patricia Moulin, Sophie Sarrassat, Marie-Paule Carlotti, Richard Lalou, Jean-Yves Le Hesran

**Affiliations:** 1 Institut de Recherche pour le Développement, UR10, Faculté de pharmacie, 4 av de l'Observatoire, 75006 Paris, France; 2 Institut de Recherche pour le Développement, US 191, BP 1386, 18524 Dakar, Sénégal; 3 Institut de Médecine Tropicale du Service de Santé des Armées, UR3P, Parc du Pharo BP 46, 13998 Marseille armées, France; 4 Institut de Recherche pour le Développement, UMR151-LPED, Centre St Charles, Case 10, 13331 Marseille cedex 3, France

## Abstract

**Background:**

Although there are many methods available for measuring compliance, there is no formal gold standard. Different techniques used to measure compliance were compared among children treated by the anti-malarial amodiaquine/sulphadoxine-pyrimethamine (AQ/SP) combination therapy, in use in Senegal between 2004 and 2006.

**Methods:**

The study was carried out in 2004, in five health centres located in the Thies region (Senegal). Children who had AQ/SP prescribed for three and one day respectively at the health centre were recruited. The day following the theoretical last intake of AQ, venous blood, and urine samples were collected for anti-malarial drugs dosage. Caregivers and children above five years were interviewed concerning children's drug intake.

**Results:**

Among the children, 64.7% adhered to 80% of the prescribed dose and only 37.7% were strict full adherent to the prescription. There was 72.7% agreement between self-reported data and blood drug dosage for amodiaquine treatment. Concerning SP, results found that blood dosages were 91.4% concordant with urine tests and 90% with self-reported data based on questionnaires.

**Conclusion:**

Self-reported data could provide useful quantitative information on drug intake and administration. Under strict methodological conditions this method, easy to implement, can be used to describe patients' behaviors and their use of new anti-malarial treatment. Self-reported data is a major tool for assessing compliance in resource poor countries. Blood and urine drug dosages provide qualitative results that confirm any drug intake. Urine assays for SP could be useful to obtain public health data, for example on chemoprophylaxis among pregnant women.

## Background

In the fight against malaria, the emergence of resistance to chloroquine, but also to other drugs, such as mefloquine or sulphadoxine-pyrimethamine, directs our attention to one of the factors of resistance emergence, i.e. drug use [1]. The theoretical effectiveness of a growing number of medical interventions appears to be hindered by patients' and caregivers' behaviors concerning compliance with treatment regimens [2,3]. In order to improve therapeutic strategies, a particular interest is focused on the measurement of compliance with treatment.

This is a complex issue since there is no benchmark [4,5]. The selection of available methods--drug assays, biological markers, questionnaires, patient interview, drug package counts--depends upon the study's target and organizational constraints [4]. Some researchers give priority to biological or clinical measurement to benchmark compliance. They contend that self-reported data can be affected by different biases--*recall *bias and *good-will *bias, since the patient tends to give the expected answer [6,7]. Nevertheless, the questionnaire is the most common method for measuring compliance in developing countries; its advantage is to have only a few logistic constraints, particularly compared to biological and blood dosages, and to be less invasive [4]. A literature review conducted within the malaria context pointed out the need to combine different methods in order to validate self-reported data--for measurement of compliance -- by biological means [8]. Nearly all malaria-endemic countries have seen an unprecedented change in national drugs policies in recent years. In line with WHO recommendations, they gave up chloroquine, which was becoming ineffective and promoted artemisin-based combination therapy as first-line treatment for mild malaria. Senegal changed its malaria treatment in 2004, from chloroquine to the amodiaquine/sulphadoxine-pyrimethamine (AQ/SP) combination before the implementation of artemisinin combination therapy (ACT) in 2006. The introduction of this combination therapy can be considered as a test before the implementation of ACT in terms of health staff behavior and public compliance with new drugs and drug combinations. At that time in Senegal, they were not provided in a single blister. These new combinations used two different drugs--AQ and SP--requiring different "dosage regimens", for three and one day respectively. This treatment regimen may have been difficult for the population to understand and thus have reduced compliance.

In a study on the measurement of compliance with these new treatments and their effectiveness, three techniques were used: self-reporting based on questionnaires, measurement of drug concentration in urine and in blood. Results were compared and the reliability, advantages, and disadvantages of each technique were assessed.

## Methods

### Study area

The study was conducted in five health facilities located in rural areas of the health districts of Mbour and Thies, 70 km southeast of Dakar. Malaria is endemic in the study area with seasonal outbreaks. The annual rainy season is short (from July to October). Malaria transmission is mainly restricted from August to November. The study took place from September to November 2004, less than a year after the implementation of combination AQ/SP in Senegal.

### Study design

During the study period, nurses identified all the children, aged between two to ten, who presented with fever (axillary temperature > 37.5°C) and a presumptive diagnosis of mild malaria during an initial consultation. Each provider was given a chart showing the appropriate dosage by weight and age, defined by the Senegalese National Malaria Control Program (Table 1). This program recommended that all children be prescribed a single dose of SP (25 mg sulphadoxine/kg; 1.25 mg pyrimethamine/kg) on day 0 (D0), and one daily dose of AQ for 3 days (D0 to D2: 10 mg/kg/day). The program did not expect nurses to supervise the first intake. Drugs were available at the health centre pharmacy.

**Table 1:** Age-specific dosing schedule for sulphadoxine-pyrimethamine (SP) and amodiaquine (AQ) in Senegal

		SP tablets*	AQ tablets**		
Age group	Weight	Day 0	Day 0	Day 1	Day 2
2--11 months	5 - 10 kg	0.5	0.5 or 2 tsp	0.5 or 2 tsp	0.5 or 2 tsp
1--2 years	10.1 -14 kg	0.75	0.75 or 3 tsp	0.75 or 3 tsp	0.75 or 3 tsp
3- 5 years	14.1 - 20 kg	1	1 or 4 tsp	1 or 4 tsp	1 or 4 tsp
6-8 years	20,1 - 30 kg	1.5	1.5	1.5	1.5
9-11 years	30,1 - 40 kg	2	2	2	2
12-13 years	40,1 - 50 kg	2.5	2.5	2.5	2.5
> 14 years	> 50 kg	3	3	3	3

* SP: 1 tablet contains 500 mg of sulphadoxine and 25 mg of pyrimethamine

** AQ: 1 tablet contains 153 mg of amodiaquine and 1 teaspoon (tsp = 5 ml) contains 50 mg of amodiaquine

A thick blood film was used to measure the parasitaemia at D0. This allowed a biological confirmation of the diagnosis and the inclusion of children in the effectiveness study. Nurses did not know the results at the time of prescription. No study team member was present at the health center. Each evening, identification forms and blood smears were collected from the nurses. Children were recruited the day after the last treatment dose (D3), when mothers/guardians were visited at home where the study design was explained and their written consent obtained. Free and informed consent was sought from the children's caregivers before administration of the questionnaires and blood samples. Apart from the study team, no one was aware of this visit. Pharmacy registers were checked regarding the dispensing of drugs to patients, the quantity of drug dispensed and the date of drug purchase to determine D3. The study was approved by the Senegalese ethical committee.

### Measurement of compliance using self-reported data

The questionnaire was addressed to caregivers and accompanying adults the day following the end-of-treatment intake (D3) or during the following three days in case of absence (D4-D5). Questionnaires were translated into the local languages; they were administered in the form of an interview. Interviewers were trained during one week for the questionnaire and a pre-test was done in villages outside the study area. The individual questionnaire included nine sections with questions related to the following issues: respondents' socio-economic and demographic characteristics, history of febrile illness, consultation at the health center, child health status between D0 and the interview day, compliance with treatment, knowledge about malaria, source of information and attitudes towards health and drugs. If the caregiver was different from the accompanying adult, he was asked the questions concerning the health center and consultation. The last section was addressed to children; they were asked about the drugs (intake, taste) and about their disease and recovery. Open questions were asked about treatment compliance including dose, duration and frequency. Other questions addressed vomiting and dose replacement. To measure compliance, the caregiver's administration of the drugs was compared with the nurse's prescription. Three levels of compliance were defined. The first one based on drug administration indicates whether there has been at least one intake of each drug. Secondly, those who took 80% of the prescribed dose of each one of the two drugs were considered as compliant [9]. The entire declared drug intake was added up and compared with the total dose prescribed by the nurse. Thirdly, strict full adherence (SFA) described a patient as fully adherent to the prescription, including dose, duration and frequency. Patients were required to take the daily exact dose of AQ prescribed for three days and the exact dose of SP prescribed on the first day. The first level --at least one drug intake--was used for comparison with the blood and urine assay results. The families were re-interviewed the month following the end of the survey, in case of discrepancies observed between self-reported and urine assay results.

### Biological assays

Biological assays were carried out among a subgroup of children. Those who presented with a biologically-confirmed malaria attack (D0 parasitaemia > 2500/μL) and who where included into the effectiveness study were recruited. Urine and blood assays (taken from inside arm at elbow) were performed to measure drug intake. Blood samples were stored at -20°C.

An assay of sulphadoxine--standing for sulphadoxine/pyrimethamine combination--was performed using a colorimetric method for detecting sulphonamides in urine. The method uses the color agent, p-dimethylaminocinnamaldehyde, and has a detection limit of about 1 μg/ml [10]. Blood samples (2 ml) were analysed to detect the presence and concentration of mono-desethyl amodiaquine (MDA), sulphadoxine, pyrimethamine, and chloroquine in order to assess a possible self-medication. A High Performance Liquid Chromatography (HPLC) method in the reversed-phase mode with an ultraviolet diode array detection (λ = 343 nm for AQ and CQ and λ = 270 nm for SP) was performed to measure molecular compounds in blood. It was based on the method developed by Pussard and Verdier [11].

## Results

### Socio-demographic characteristics of children and compliance

The study was conducted in five health centers in which 289 children were recruited. One hundred and forty-five children had laboratory-confirmed malaria (parasitaemia > 2,500/μl) and 144 were diagnosed presumptively by the nurses (117 were negative and 28 had a parasitaemia < = 2,500/μl). The median age of the recruited children was 5.4 years. The sex ratio (male/female) was 1.3. The mother was the caregiver in 69.5% of the time followed by the grandmother (13.2%), the father (7.6%) and others (9.7%). Among all recruited children, 204 of them (70.6%) were administered AQ in tablet form and 85 (29.4%) in syrup form. Two caregivers (0.7%) did not buy AQ and six (2.1%) did not buy SP.

Among the 145 children presenting with malaria--validated by a biological test--some 138 (95.2%) venous blood samples for an assay of amodiaquine and SP and 139 urine samples (95.9%) for an assay of sulphadoxine were performed at D3. All caregivers of the 289 recruited children were interviewed using a questionnaire between D3 and D5.

### Measurement of compliance

#### Self-reported data

Most patients (94.1%) took at least one dose of each of the two drugs. Among the children, 64.7% were considered as compliant to the dose prescribed by the nurse, and 37.7% were fully compliant to the prescription of the two medications. Compliance was higher for SP than for AQ (Table 2). Strict full adherence to amodiaquine was higher among children who were administered tablets (45%) than among those who took syrup (28.2%).

**Table 2:** Adherence indicators: results of self reported data

	AQ	SP	Combination
Intake	285 (98.6%)	272 (94.1%)	272 (94.1%)
Adherent (with 80% threshold)	209 (72.3%)	245 (84.8%)	187 (64.7%)
Strict full adherent	114 (39.4%)	176 (60.9%)	109 (37.7%)

**Intake**: administration of the drug at least one time; **Adherent (with 80% threshold)**: administration of 80% of the prescribed dose; **Strict Full Adherence**: administration exactly as prescribed by the nurse (dose, duration, frequency)

#### Blood and urine assays

Results obtained with HPLC indicate that a concentration of the amodiaquine metabolite--mono-desethyl amodiaquine (MDA)--has been measured in blood for only 70.5% of cases. Regarding SP, 92.8% of patients had positive blood dosages of both sulphadoxine and pyrimethamine that express a double confirmation of drug intake. Some 84.2% of children had a positive urine test for sulfonamides (Table 3).

**Table 3:** Comparison of results based on self-reported data and urine assays with blood assays

		SP assays in blood		
		Positive	Negative	Total
Self-reported data/SP intake	Positive	122 (87.8%)	7 (5%)	129 (92.8%)
	Negative	7 (5%)	3 (2.2%)	10 (7.2%)
	Total	129 (92.8%)	10 (7.2%)	139 (100%)

SP assays in urine	Positive	117 (84.2%)	0	117 (84.2%)
	Negative	12 (8.6%)	10 (7.2%)	22 (15.8%)
	Total	129 (92.8%)	10 (7.2%)	139 (100%)

				
		**AQ assays in blood**		
		**Positive**	**Negative**	**Total**

Self-reported data/AQ intake	Positive	98 (70.5%)	38 (27.3%)	136 (97.8%)
	Negative	0	3 (2.2%)	3 (2.2%)
	Total	98 (70.5%)	41 (29.5%)	139 (100%)

### Comparison between results based on different methods

#### Sulphadoxine assays in blood and urine

There is good correlation between blood and urine dosages for SP: for 91.4% of patients, identical results were found using both techniques. Using the HPLC assay method as the reference, no false positives were observed and the rate of false negatives was 8.6% (12/139). The mean concentration using HPLC was 25.3 μg/ml [9.4-41.3] for patients with a negative urine test (22/139) and 74.8 μg/ml [66.2-79.5] for those with a positive urine test (117/139) (p < 0.001) (Table 3).

#### Sulphadoxine assays in blood and self-reported data

There was a 90% agreement between data from questionnaires and results obtained by HPLC. Seven caregivers reported having administered SP to their children while dosages by HPLC were negative and seven others said they did not give SP to their children while HPLC dosages were positive--i.e. 5% of false positives and 5% of false negatives (Table 3).

#### Blood dosages of AQ and self-reported data

The correspondence between blood dosages and questionnaires responses was 72.7%. Many patients had a negative dosage by HPLC--41 patients among 139, i.e. 29.5%. Among these patients, only three reported not having taken amodiaquine (Table 3). The mean duration between the last intake and the sample was similar for both groups--10.6 hours [8.9-12.5].

#### Reported intake and concentration in blood or urine

**Figure 1:**
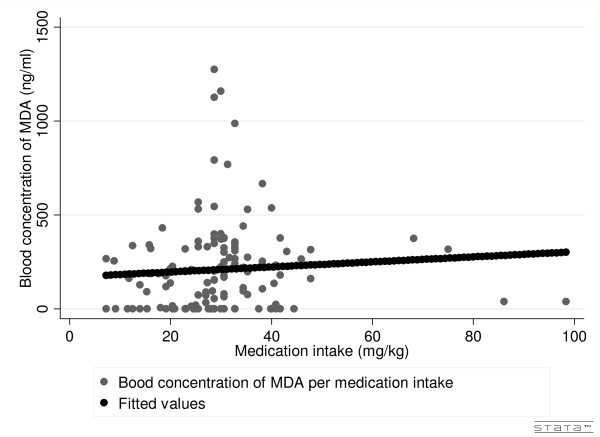
**Blood concentration of monodesethylamodiaquine by self-reported medication intake**.

**Figure 2:**
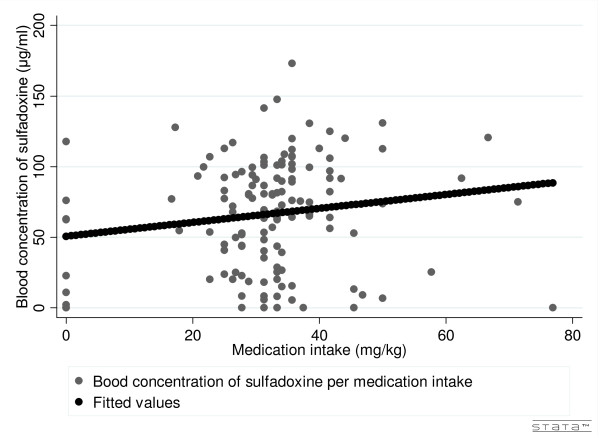
**Blood concentration of sulphadoxine by self-reported medication intake**.

For SP, concentrations of sulphadoxine in blood and urine were used and for amodiaquine, blood concentrations of its metabolite--mono-desethylamodiaquine (MDA). The results do not allow us to establish a relationship between the quantity of medication intake (mg/kg) and the molecule concentrations found in blood (Figure 1 and 2). Correlation was respectively 0.1606 and 0.1591 for S and P and 0.0704 for AQ; results were insignificant (0.0628, 0.0654 and 0.4204).

## Discussion

### Amodiaquineassays in blood: encountered storage difficulties

For almost one third of children (29.5%), the blood concentration of MDA was nil, while their parents had reported giving them amodiaquine. A low rate of positive assays seems to be related to the conservation of the sample. The assays have been performed two years after the collection. In this study, glass tubes were used, whereas polypropylene tubes would probably have limited the adsorption of the MDA molecule by the glass [12]. Given the literature, this seems to be a potential argument. The last hypothesis concerns the preservation of samples in terms of their storage in a dark place to prevent photo-degradation of the molecule and its metabolites [13], and the temperature to preserve samples. In a recent study, tubes of spiked blood containing AQ and MDA were kept respectively at -20°C and -86°C for 148 days. Only 35% of the initial quantity of AQ was found in the whole-blood samples that were kept at -20°C, whereas 97% to 99% were measured in the whole-blood samples kept at -86°C and in stored plasma kept at -20°C or -86°C [14].

### SP assays in blood: a good correlation between results

Each blood sample was tested for both sulphadoxine and pyrimethamine. Results were similar for all the subjects. In addition, a good correlation with results based on self-reported data was observed. The cases of discrepancies--seven false negatives and seven false positives--were examined and are explained by the fact that families had difficulties identifying the drugs once the blisters were cut and put in a bag. The SP tablet--white and approximately the size of an aspirin tablet--was identified as an aspirin tablet and families reported having only taken an antipyretic. Conversely, other families reported having given SP though they only gave aspirin. The observed differences seem to be explained more by a confusion between the drugs than by the respondent's desire to conceal information.

### Advantages and disadvantages of blood assays

Venous blood sampling has the disadvantage of being invasive and often badly perceived by families, in particular when it concerns young children [15]. However, in the study there were no refusals to perform blood tests, but there were missing samples as a result of absence of children at D3 and some failures due to the difficulty of some samplings (4.8%). This success is the result of the establishment of a close relationship with the families and community leaders. This method is also restrictive in terms of time--time-length between medication intake and blood sampling as well as between sampling and freezing-- and sample preservation particularly in the field where appropriate material conditions are not always available. Moreover, the results show that blood assays only give qualitative information on whether there has been any drug intake. A strong variability of blood concentration whatever the intake has been observed [16]. This high inter-individual variability of MDA could be attributed to the different rates of hepatic metabolism of each individual. It appears in others surveys to have a greater effect on MDA levels than the number of timing doses [17,18]. Blood assays seem to have a limited role in discussing drug intake after no intake or intake of one and more doses [17].

### SP assays: the advantages of urine over blood samples

Results from urine dosages of sulphadoxine correspond with the blood dosage results. The sensitivity of this measure is 91% and the specificity is 100%. In his study, Mount obtained a sensitivity of 94% and a specificity of 94% while comparing the results from this method with those using HPLC [10]. This method is particularly appealing given the good sensitivity and specificity of the assays as well as the simplicity of use and ease in sampling compared to a venous blood sample. SP is currently no longer or rarely used in artemisinin-based combination therapy. Nevertheless, it is used in the form of intermittent preventive treatment (IPT) for pregnant women for malaria chemoprophylaxis. Urine samples are often collected among pregnant women during prenatal care to measure albumin. This technique of urine assays could easily be implemented, and used for studies on compliance and effectiveness of this prophylaxis during uncontrolled intakes. Results such as those obtained with blood assays only provide a qualitative validation of the intake. In this case, the purpose is not to collect individual data but to have an interesting epidemiological tool to obtain public health data on chemoprophylaxis among pregnant women.

### Validity of questionnaires

Self-reported data on medication intake are often controversial. Self-reported data have the disadvantage of being subject to the reliability of the respondent's self-reporting of his or her behavior. There is also a potential recall bias, inherent in all data collection subsequent to the events [19,6]. In a study carried out in Malawi regarding a prior treatment to the examination at the health centre, families reported not having taken tablets, although he found sulphadoxine or chloroquine in the children's urine [7]. In another study, conducted on compliance with chemoprophylaxis among pregnant women, the reported compliance was much higher than the one observed by assays [20]. In this case, it is mentioned that the bias stems from the fact that subjects are prone to answer what is expected of them [21]. Conversely, other studies demonstrate a good reliability of results based on self-reporting. In a study carried out in China, Qingjun found a 97% compliance with a primaquine and chloroquine blister based on self-reporting and on urine assay of phenobarbitol--added as a drug marker [22]. In a study in Uganda [23], compliance was measured by self-reporting, blister counts, and plasma lumefantrine assay. Fogg found lower levels of lumefantrine in plasma among non-adherent patients than among adherent patients, although the results were not statistically significant [23].

A good correlation was found between self-reported data and SP assays by HPLC. These results validate the value ascribed to self-reported data whenever patients did not experience this as a means to check their behaviors [3]. The interviewer must establish a relationship with the patient without expressing any judgment or attaching any value to the respondent's answers [3]. A long and detailed interview with the child's caregiver is needed to obtain reliable data and reconstruct the drug-intake history. The one-hour long interview may have appeared as a constraint for some families. However, there were no refusals. The quality of the questioning is also essential. The questionnaire is a specific scientific tool for which the structure and sequences of questions are capital. The quality of results will depend on these components. Internal validation methods exist that use cross-questions to specifically validate responses. The delay between the events and collection of data is also a central element to limit recall biases. The fact that interviews with the families were carried out the days following the end of the treatment restricted recall biases. Even under strict methodological conditions, the collection of self-reported data in the field is less constraining than biological assays. Questionnaires were filled out between the third and fifth day after the medical examination; this simplified the material organization of the study. Furthermore, self-reported data provided in-depth information on children's drug use in terms of timing, amount, and duration. Another method would not have provided such accuracy. These data have also highlighted the patients' difficulties and behaviors concerning drug intake and compliance with the treatment, and allow us to better understand the factors potentially involved [24].

## Conclusion

Compliance is not only the measurement of medication intake, but also concerns the intake timing, doses, and treatment duration. All these dimensions can only be assessed by the use of self-reported data or by the implementation of more expensive modern methods such as electronic pill-boxes. This confirms the specific contribution of self-reported data to measure compliance. Blood and urine drug dosages provide qualitative results useful to confirm drug intake. Urine assays for SP could be useful to obtain public health data, like for chemoprophylaxis among pregnant women. In malaria-endemic countries self-reported data appears under rigorous methodology conditions --e.g. using confirmation by hospital prescription records-- as the easiest method to comprehend the patients' behavior and drug use in order to develop tools to improve compliance with treatment regimens.

## Competing interests

The authors declare that they have no competing interests.

## Authors' contributions

AS: conception and design of the study, data collection, analysis and interpretation of data, drafting the paper; PM, SS, MPC: study implementation, data collection, analysis and interpretation of the data; RL and JYLH: conception and design of the study, analysis and interpretation of data; all authors revised the article critically and approved the final manuscript; AS and JYLH are guarantors of the paper.
